# Estimating the effects of COVID-19 on essential health services utilization in Uganda and Bangladesh using data from routine health information systems

**DOI:** 10.3389/fpubh.2023.1129581

**Published:** 2023-09-27

**Authors:** Gustavo Angeles, Hannah Silverstein, Karar Zunaid Ahsan, Mohammad Golam Kibria, Nibras Ar Rakib, Gabriela Escudero, Kavita Singh, Jamiru Mpiima, Elizabeth Simmons, William Weiss

**Affiliations:** ^1^Department of Maternal and Child Health, Gillings School of Global Public Health, University of North Carolina at Chapel Hill, Chapel Hill, NC, United States; ^2^Public Health Leadership Program, Gillings School of Global Public Health, University of North Carolina at Chapel Hill, Chapel Hill, NC, United States; ^3^Carolina Health Informatics Program, Gillings School of Global Public Health, University of North Carolina at Chapel Hill, Chapel Hill, NC, United States; ^4^Carolina Population Center, University of North Carolina at Chapel Hill, Chapel Hill, NC, United States; ^5^Last Mile Health–Uganda, Kampala, Uganda; ^6^Department of International Health, Johns Hopkins University, Baltimore, MD, United States

**Keywords:** SARS-CoV-2, coronavirus disease 2019 (COVID-19), maternal and child health, health services use, routine health information system, time series model

## Abstract

**Background:**

Since March 2020, the coronavirus disease 2019 (COVID-19) pandemic has been a major shock to health systems across the world. We examined national usage patterns for selected basic, essential health services, before and during the COVID-19 pandemic in Uganda and Bangladesh, to determine whether COVID-19 affected reporting of service utilization and the use of health services in each country.

**Methods:**

We used routine health information system data since January 2017 to analyze reporting and service utilization patterns for a variety of health services. Using time series models to replicate pre-COVID-19 trajectories over time we estimated what levels would have been observed if COVID-19 had not occurred during the pandemic months, starting in March 2020. The difference between the observed and predicted levels is the COVID-19 effect on health services.

**Results:**

The time trend models for Uganda and Bangladesh closely replicated the levels and trajectories of service utilization during the 38 months prior to the COVID-19 pandemic. Our results indicate that COVID-19 had severe effects across all services, particularly during the first months of the pandemic, but COVID-19 impacts on health services and subsequent recovery varied by service type. In general, recovery to expected levels was slow and incomplete across the most affected services.

**Conclusion:**

Our analytical approach based on national information system data could be very useful as a form of surveillance for health services disruptions from any cause leading to rapid responses from health service managers and policymakers.

## 1. Background

On March 11, 2020, the World Health Organization (WHO) characterized the coronavirus disease 2019 (COVID-19) outbreak as a pandemic (1). With cases reported in more than 110 countries and territories at the time (2), countries took steps to attempt to prevent further spread by resorting to social distancing, closing of schools and businesses, and implementing lockdowns. Since March 2020, there have been countless ways the COVID-19 pandemic has directly and indirectly affected societal structure and function, from movement restrictions to personnel shortages, and global supply chain interruptions.

The COVID-19 pandemic and the countries’ response delivered a massive shock to the supply and demand of goods and services in the entire economy. These also disrupted the availability, access, and use of health services unrelated to COVID-19. A scoping review that summarizes existing work on the effects of COVID-19 on other essential health services found that most research in this area focuses on high-income countries (3). Several studies found that COVID-19 had negative effects on health service use in low-and middle-income countries (4–11). Particularly in South Asia and Sub-Saharan Africa, a significant reduction in service utilization was noted for all essential health services, including maternal and child health services, during the first and second waves of the outbreak of COVID-19 (7, 12–15).

However, these effects have not necessarily been evenly distributed across all health services, facilities, or geographies. In India and Pakistan, studies using routine health information systems (RHIS) documented up to 82% reduction in childhood pneumonia treatment and 57% reduction in cesarean deliveries (14, 15). On the contrary, studies using RHIS in Ethiopia and South Africa noted significant reductions in child health services while maternal health services were less affected (16, 17). Studies by Ayele and colleagues and Hategeka and colleagues found that COVID-19 had negative impacts on illness-and disease-specific utilization (4, 6), but effects on vaccinations as well as maternal and infant health services were not consistent (4–7, 18). For example, Shapira and colleagues found that while several countries experienced significant decreases in the utilization of maternal and child health services (MCH), including vaccinations, the magnitude and timing of COVID-19 effects varied across countries (7). Ayele and colleagues and Hategeka and colleagues also found no difference in service utilization for selected MCH services in Addis Ababa and Kinshasa, respectively, whereas Wanyana and colleagues found a significant negative impact of COVID-19 in Rwanda (4, 6, 18). These studies are all important contributions for further understanding the extent of COVID-19 interruptions to health systems. Yet, there are still some challenges that remain when trying to interpret the results across systems. There is heterogeneity in methodological strategies, services selected for analysis, and pre-COVID-19 timeframes for estimating effects during the COVID-19 period that makes ascertaining whether inconsistent COVID-19 effects across countries reflects country differences or methodological differences. Some studies have opted for descriptive analyses, comparing 2019 pre-COVID-19 months to 2020 COVID-19 months (4, 12, 18, 19). Other researchers estimated changes in time trends before and during COVID-19 (5, 6), or used time trend models with unique variables to estimate month-specific COVID-19 effects (7, 8).

Additionally, especially during the initial lockdown periods, it is difficult to tell at which level negative effects of COVID-19 occurred. There could have been challenges to the information system, such as outages, that could have affected the quality of reporting. Under this circumstance, COVID-19 effects on utilization could be misattributed to reduced patient demand when, in fact, such effects could be caused by lower reporting. Shapira and colleagues addressed both of these issues by applying the same analytical strategy for eight countries in Sub-Saharan Africa, which includes investigations on facility-level information system reporting (7). In addition, several published studies have analyzed a relatively short time frame, only looking at COVID-19 effects through mid-2020 (4, 6–8, 18, 19), which is clearly important, given the need to provide information about a major crisis. As the pandemic has been ongoing, the effects on health services may evolve over time as well. Such information is important for policy decisions.

Both Uganda and Bangladesh follow a hierarchical structure to provide primary, secondary, and tertiary healthcare services to the population. In Uganda, community-level health services are provided through village health teams and health centers (types I–IV) as well as general hospitals situated at the district level provide essential primary health care (20). In Bangladesh, community-level health services are provided through community clinics, and essential primary health care services are provided up to the sub-district (upazila) level health facilities (21). In both countries, essential primary health care services involve general outpatient services, maternal and child health services (e.g., immunization, antenatal and delivery care), basic laboratory services, treatment for common diseases such as malaria, diarrhea, and respiratory infections, and referrals to higher levels of care. The Data for Impact (D4I) project, led by a team from the University of North Carolina at Chapel Hill through the Carolina Population Center in partnership with Palladium, ICF, John Snow, Inc. (JSI), and Tulane University, is working in Uganda and Bangladesh to strengthen the collection, management, and use of quality RHIS (22, 23). Based on our existing collaboration with governmental as well as non-governmental organizations that facilitated data access and preparations at the national level, we worked with government agencies in these countries to access the national routine data on service delivery and gathered the available datasets for analysis. This allowed us to use routine health data with national coverage for analysis, where data from all districts in Uganda and all sub-districts in Bangladesh can be used. To examine the effect of COVID-19 on health services use, we focused on essential maternal and child health services as identified by government documents and policies, as well as WHO’s core indicators of women and children’s health (24, 25). This study, therefore, estimates the effects of COVID-19 in Uganda and Bangladesh, on selected non-COVID-19-related health services using RHIS data collected prior to COVID-19 and throughout the first year of the pandemic. The two main objectives of this research are to determine whether the COVID-19 pandemic affected (1) reporting of health service utilization, and (2) utilization of health services.

## 2. Methods

### 2.1. Data

This study uses data available through the existing RHIS in each country (see basic information in Table 1). We worked with government agencies to access the government’s routine data on service delivery and gathered the available datasets for analysis. We examined 11 basic health services for Uganda and eight for Bangladesh, based on WHO guidance on the measurement of women and children’s health (24) and identified as essential services by the respective governments. The available data consisted of monthly aggregated service volumes provided at the upazila (or subdistrict) level in Bangladesh and at the district level in Uganda. Because the data were volume aggregates, they did not have any personal identifiers. Data were compiled monthly starting in January 2017. We used 3 years before COVID-19 to better identify any seasonality patterns in service use. A specific cleaning process was applied to all data prior to analysis to identify and remove outliers. For each health service, unit-specific (district for Uganda, upazila for Bangladesh) time series graphs were generated to identify extreme values. We then calculated the mean and standard deviation for each unit. The graphs and summary statistics helped to inform rules by which to remove outliers, such as removing values that exceeded five or six standard deviations above the unit-specific mean. The choice of outlier cutoff was made by examining the time trajectories of the specific units where outliers were suspected and their relative volatility over time. Such rules were adjusted based on service and country. Less than 0.2% of data points for each service within each country were deemed outliers.

**Table 1:** Dataset description.

Country	RHIS system used	Data unit-level	Time range	Health services
Uganda	District Health Information System (DHIS2)	District (*N* = 136)	January 2017–May 2021	New outpatient visits (overall attendance) 1st antenatal visits 4th antenatal visits Facility deliveries Family planning visits* Diarrhea cases (28 days–5 years) Malaria cases (28 days–5 years) Pneumonia cases (28 days–5 years) BCG doses Measles doses 1st DPT doses
Bangladesh	Management Information System 3 (MIS3) Expanded Program on Immunization (EPI)	Upazila (*N* = 489)	January 2017–August 2020	Outpatient visits (overall attendance) 1st antenatal visits 4th antenatal visits Facility deliveries Family planning**
		Upazila health complex (*N* = 424)	January 2017–April 2021	BCG Measles-rubella doses 1st pentavalent

RHIS: Routine health information system; BCG: Bacillus Calmette–Guérin vaccine; DPT: diphtheria, pertussis, and tetanus toxoids vaccine. *Uganda family planning data is a composite of the most common methods that were consistent across time, which include male and female condoms, injectables, implants, and oral contraceptives. Other methods such as IUDs, emergency contraceptives, and natural methods, were not included. **Bangladesh family planning data available ends in July 2020.

### 2.2. Measures of service use

The basic health services included in this analysis fall broadly in four categories: maternal and child health (first and fourth antenatal care [ANC1 and ANC4] visits, facility deliveries, common childhood illnesses); childhood immunization (BCG, measles, and DPT/pentavalent vaccination); family planning visits; and outpatient attendance to capture the overall service utilization (see Table 1).

For each service analyzed, to answer the first research question (i.e., did COVID-19 affect reporting?), we generated variables that counted the number of units reporting each month. For the second question (i.e., did COVID-19 affect utilization of health services?), we examined two measures of use: total services provided, and average services provided each month included in the analysis.

### 2.3. Analytical approach

The overall strategy for estimating COVID-19 effects is an adaptation of the interrupted time series approach. There were two stages in analyzing the data. The goal of the first stage was to examine pre-COVID-19 patterns and fit a time trends model that most closely replicated those observed pre-COVID-19 trajectories of service utilization over time. The goal of the second stage was to estimate what levels of service use would have been observed had COVID-19 not occurred during the COVID-19 months. To do this, we extended the models fitted to pre-COVID-19 patterns from the first process through the COVID-19 period by generating predicted values. The difference between the observed and the predicted levels for each month of the pandemic is the “COVID-19 effect.” Because each country first reported COVID-19 cases in March 2020 and experienced subsequent shutdowns/restrictions in movement, the COVID-19 period goes from March 2020 onward. Time between January 2017 and February 2020 was defined as the pre-COVID-19 period. All methods in this study were carried out using aggregated data, for which no informed consent was required, and the University of North Carolina at Chapel Hill’s Office of Human Research Ethics (OHRE) determined that this study did not constitute human subjects research as defined under federal regulations 45 CFR 46.102 (e or l) and 21 CFR 56.102(c)(e)(l). After review, the OHRE determined that this study did not require an Institutional Review Board (IRB) approval.

#### 2.3.1. Descriptive analysis and examining pre-COVID-19 patterns

We used descriptive analysis to answer the first question: did COVID-19 affect reporting? We counted the number of units reporting in each time point for each service and examined any variation over time using graphs. To answer our first research question, we examined the number of districts and upazilas reporting across the study period for each service indicator. Examining reporting over time was important for understanding reporting seasonality and changes to the health information systems relative to potential changes due to COVID-19. In countries with marked changes in climatic conditions, existing studies show that seasonal pattern exists between disease incidence (e.g., malaria, cholera, respiratory diseases) or health outcome and utilization of MCH services (26–28).

Moreover, the Management Information System 3 (MIS3) system expanded in 2019 in Bangladesh, which posed challenges for modeling sub-national geographic stratifications for the capital Dhaka. Two of the larger sub-districts in Dhaka, which accounted for a substantial proportion of the total service utilization, had serious issues with completeness and reliability of data during the expansion period (whereas the system expansion did not seem to affect the observed service utilization levels in other sub-districts). For these reasons, replicating pre-COVID patterns was challenging for Dhaka district, and had we imposed pre-COVID trajectories for Dhaka during the COVID period, that would have been misleading. So, we decided to keep the entire analysis only at the national level for Bangladesh.

Time trends model estimation procedures were used to answer the second question: did COVID-19 affect service utilization? For each service, several tests and pieces of information were used to make final determinations on which model to use for estimating the COVID-19 effect. In particular, we undertook several steps to determine which functional form of time better replicated the trajectories of service utilization before COVID-19 (detailed in Supplementary material).

#### 2.3.2. Replicating pre-COVID-19 service use patterns and estimating COVID-19 effects

We estimated various regression models to best replicate the observed levels and trajectories of each service indicator prior to the pandemic for each country. We conducted an initial investigation to identify the best functional form for these models. The functional form analysis indicated that most services followed a linear time trend for both countries. However, to confirm these conclusions, we also estimated models with quadratic specifications of time. For each functional form, we explored a variety of estimation approaches, including ordinary least squares (OLS), fixed effects, random effects, and autoregressive models. All models examined included seasonal (i.e., quarterly or monthly dummy variables) and yearly controls. For estimating the COVID-19 effect on total service use we included the total number of units reporting the service as a regressor in the model. More details on the model fit assessment can be found in Supplementary material.

From the functional form analysis, we determined linear OLS models with monthly seasonal controls best replicated pre-COVID-19 trajectories of total service utilization in both countries. We used the following specification:


(1) $$\begin{array}{l} \text{TotalUse}K_{t}=\alpha_{0}+\alpha_{1}time_{t}+\mathrm{\beta Mont}h_{t}\\ +\mathrm{\gamma yea}r_{t}+\mathrm{\delta NUnits}K_{t}+\varepsilon_{t},\text{for}\;t\leq 38 \end{array}$$

where $\text{TotalUse}K_{t}$ is the number of total services reported as provided in month t during the pre-COVID-19 period (January 2017–February 2020; t≤38) for service *K*; $time_{t}$ is the number of months since January 2017 up to t; $\text{Mont}h_{t}$ is a set of monthly dummy variables to control for seasonality; $year_{t}$ is a set of yearly dummy variables; $\text{NUnits}K_{t}$ is the number of units reporting service *K* in month t; and $\varepsilon_{t}$ is the error term.

We then used estimated model (1) to obtain predicted total service use values had COVID-19 not occurred during the COVID-19 period ($\text{Tot}\overset{⏜}{alU}seK_{t}$, for t≥39), and estimated the COVID-19 effect with the following expression:


$$\text{COVID}-19\;\text{Effec}t_{t}=\text{TotalUse}K_{t}-Tol\overset{⏜}{alU}seK_{t},\text{for}\;t\geq 39$$

For vaccination services in Bangladesh, EPI data was available at the health facility level (the upazilla health complex). By using graphs, we observed fluctuations in the number of facilities reporting during the COVID-19 period which suggested that COVID-19 might have affected reporting of these vaccination services. To control for this indirect effect of COVID-19 on facility reporting, we also estimated reporting-adjusted COVID-19 effects on total vaccination services. We first modeled the number of health units reporting vaccination services K ($\text{NUnits}K_{t}$) in the pre-COVID-19 period as follows:


(2) $$\text{NUnits}K_{t}=\beta_{0}+\beta_{1}time_{t}+\mathrm{\pi Mont}h_{t}+\mathrm{\tau Yea}r_{t}+\varepsilon_{t},\;\text{for}\;t\leq 38$$

and then we obtained the predicted number of health units that would have reported vaccination service K had COVID-19 not occurred from March 2020 onwards ($N\overset{⌢}{\text{Units}}K_{t}$, t≥39). We used these predicted values to recalculate the predicted total service use using the estimated parameters from model (1):


$$\begin{array}{l} \text{Tot}\overset{⌢}{alU}seK_{t}^{\text{Adjusted}}={\overset{⌢}{\alpha }}_{0}+{\overset{⌢}{\alpha }}_{1}time_{t}+\overset{⌢}{\beta }\text{Mont}h_{t}+\overset{⌢}{\gamma }year_{t}\\ +\overset{⌢}{\delta }N\overset{⌢}{\text{Units}}K_{t},\;t\geq 39 \end{array}$$

Then, the reporting-adjusted COVID-19 effects for vaccinations are estimated with:


$$\begin{array}{l} \text{COVID}-19\;\text{Effec}t_{t}^{\text{Adjusted}}=\text{TotalUse}K_{t}-\text{Tot}\overset{⌢}{alU}seK_{t}^{\text{Adjusted}},\;\\ \text{for}\;t\geq 39 \end{array}$$

For examining average service use per reporting unit, the linear fixed effects model best replicated trajectories during the pre-COVID-19 period for Uganda and Bangladesh. We estimated the following model:


(3) $$\begin{array}{l} \text{Use}K_{jt}=\alpha_{0}+\alpha_{1}time_{t}+\mathrm{\beta Mont}h_{t}+\mathrm{\gamma Yea}r_{t}+\delta_{j}+\varepsilon_{jt},\;\\ \text{for}\;t\leq 38 \end{array}$$

where $\text{Use}K_{jt}$ is the number of services *K* provided in administrative unit j in month t; $\delta_{j}$ is a set of administrative units (i.e., district or upazila) dummy indicators included to control for time-invariant unobserved factors specific to each administrative unit that influence the demand and supply for services; and $\varepsilon_{jt}$ is the error term. Model (3) allowed us to obtain predicted average service use had COVID-19 not occurred (${\overset{⌢}{\text{UseK}}}_{t},$ for t≥39). We then estimated the COVID-19 effect on average service use with:


$$\text{COVID}-19\;\text{Effec}t_{t}=\text{Use}K_{t}-{\overset{⌢}{\text{UseK}}}_{t},\;t\geq 39$$

#### 2.3.3. Quantifying the effects of COVID-19

After deciding on the model specification for each country and service, we then used those models to predict levels of service utilization and obtained standard errors for the entire time series, including the COVID-19 period. We estimated test statistics based on the *t*-distribution for results from average utilization models and the z-distribution for total utilization models, as well as corresponding *p*-values and 95% confidence intervals to test the null hypothesis that the difference between observed and predicted values was not significantly different than zero for a given month in the COVID-19 period. If the difference between observed and predicted was significantly different from zero, then there was a COVID-19 effect in that month.

**Figure 1:**
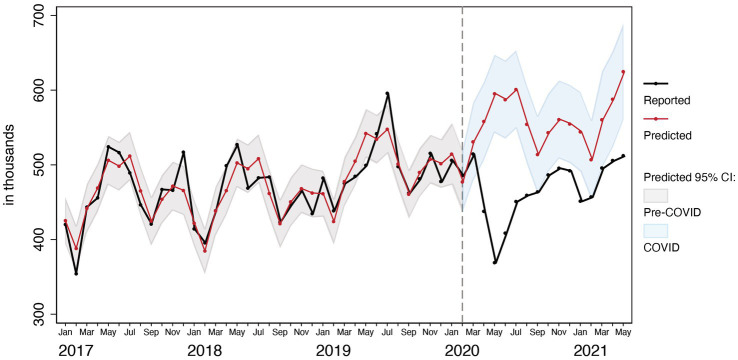
Observed and predicted total outpatient attendances visits before and after COVID-19 in Kampala Metropolitan Area.

An example of one of these models is in Figure 1, which shows observed and predicted levels of total outpatient visits in the city of Kampala. The black line is the observed number of total outpatient visits. The red line is the respective values predicted by our time trends model. The vertical dashed line at February 2020 divides the time series into the pre-and post-COVID-19 periods. Our goal was first to identify the model specifications that best replicate the trajectories observed in the area to the left of the vertical line. The model mostly replicates well the observed trajectories. The blue area represents the 95% confidence interval for the predicted values during COVID-19 months had COVID-19 not occurred. Time points where the black line extends beyond the shaded blue area are months where the observed levels do not fall within the predicted range. In Figure 1, COVID-19 significantly impacted total outpatient visits in Kampala for the entire pandemic period except March 2020, September 2020, and February 2021. The difference between the predicted and observed during the pandemic months is the COVID-19 effect.

We present COVID-19 effect estimates in terms of percentage units relative to the predicted values to assess the magnitude of the COVID-19 effect relative to the situation of service use had COVID-19 not occurred. We used the following equation:


(4) $$\text{COVID}-19\;\text{Effect}\;\text{perc}=\left(\frac{\text{Us}e_{t,\text{observed}}-{\overset{⌢}{\text{Use}}}_{t}}{{\overset{⌢}{\text{Use}}}_{t}}\right)\times 100$$

## 3. Results

### 3.1. Effects of COVID-19 on reporting

**Figure 2:**
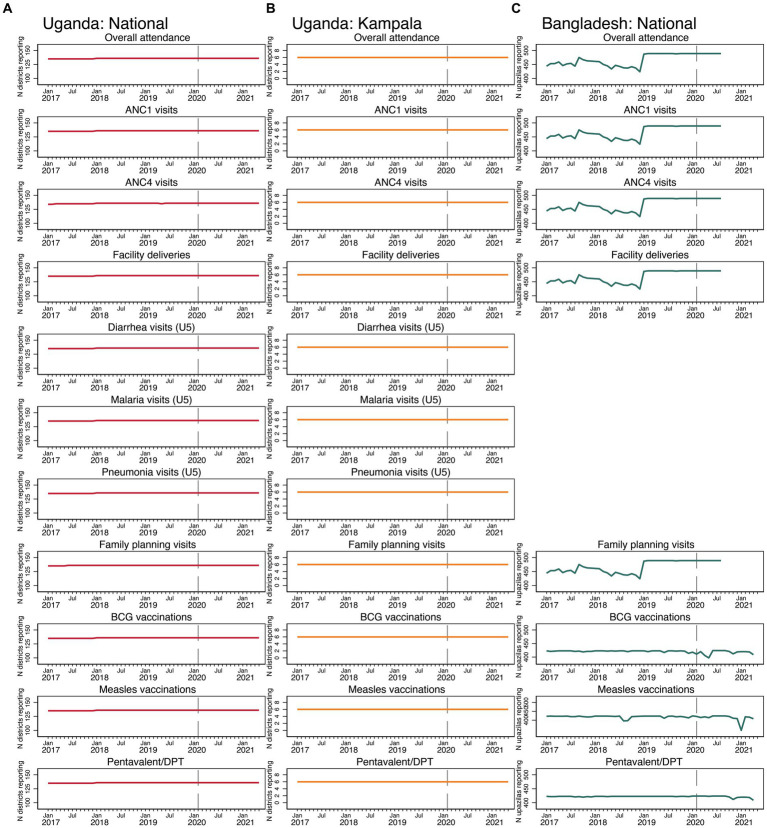
Number of reporting districts in Uganda and upazilas in Bangladesh, by service **(A)** Uganda: National, **(B)** Uganda: Kampala, and **(C)** Bangladesh: National.

Our initial descriptive analysis centered around first research question: did COVID-19 affect the reporting of services to the health information system? Figure 2 shows the number of units (districts in Uganda and upazilas in Bangladesh) reporting in each dataset, across all services. In all services in Uganda and all services in Bangladesh except for the immunization-related services, there was very little change in reporting across the time series and no change in reporting during the COVID-19 period. Therefore, there is no evidence of COVID-19 effects on reporting, except for vaccination reporting in Bangladesh.

### 3.2. Effects of COVID-19 on outpatient attendance

**Figure 3:**
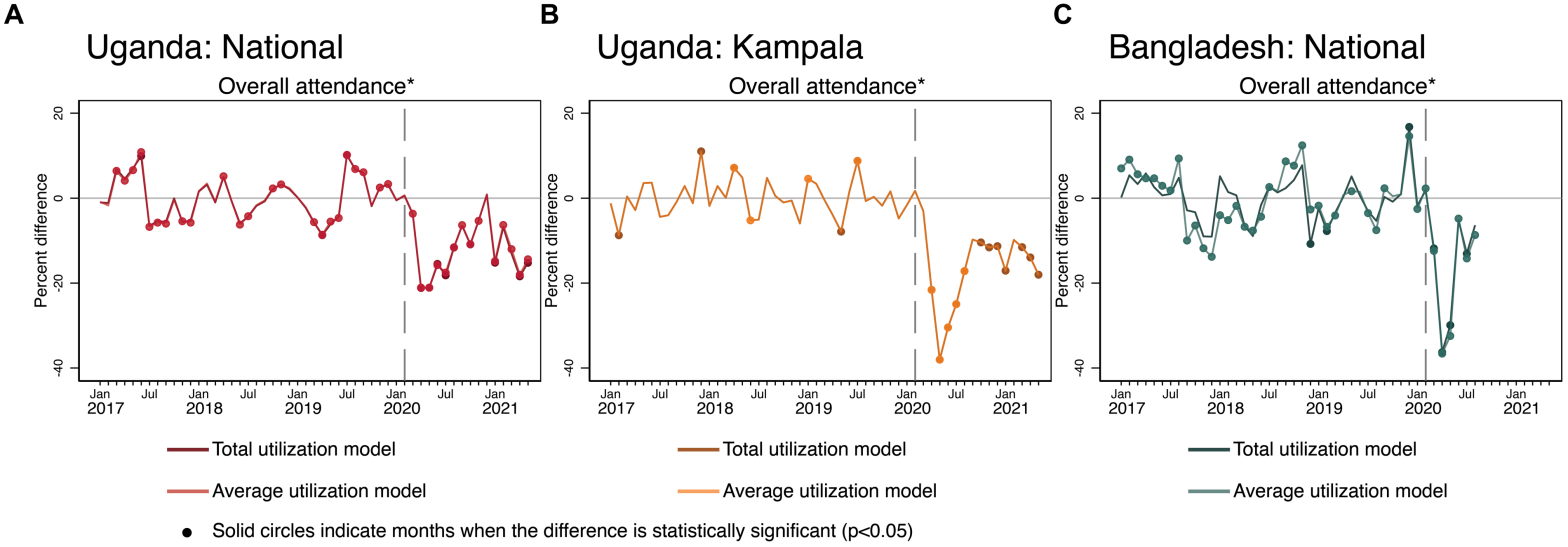
Estimated COVID-19 effects on overall outpatient attendance in Uganda and Bangladesh **(A)** Uganda: National, **(B)** Uganda: Kampala, and **(C)** Bangladesh: National. Overall attendance is measured differently across countries. In Bangladesh, overall attendance was defined as the sum of all male and female outpatient visits. In Uganda, overall attendance was the number of new outpatient visits.

There were severe negative COVID-19 effects on total and average overall attendance in Uganda and Bangladesh (see Figure 3). The COVID-19 effects on outpatient attendance both in terms of the average and totals were more severe in Kampala compared to Uganda nationally. In both countries and in Kampala, outpatient attendance largely did not recover by the end of each time series, despite some improvements after the initial months of the pandemic.

### 3.3. Effects of COVID-19 on maternal and newborn health services

**Figure 4:**
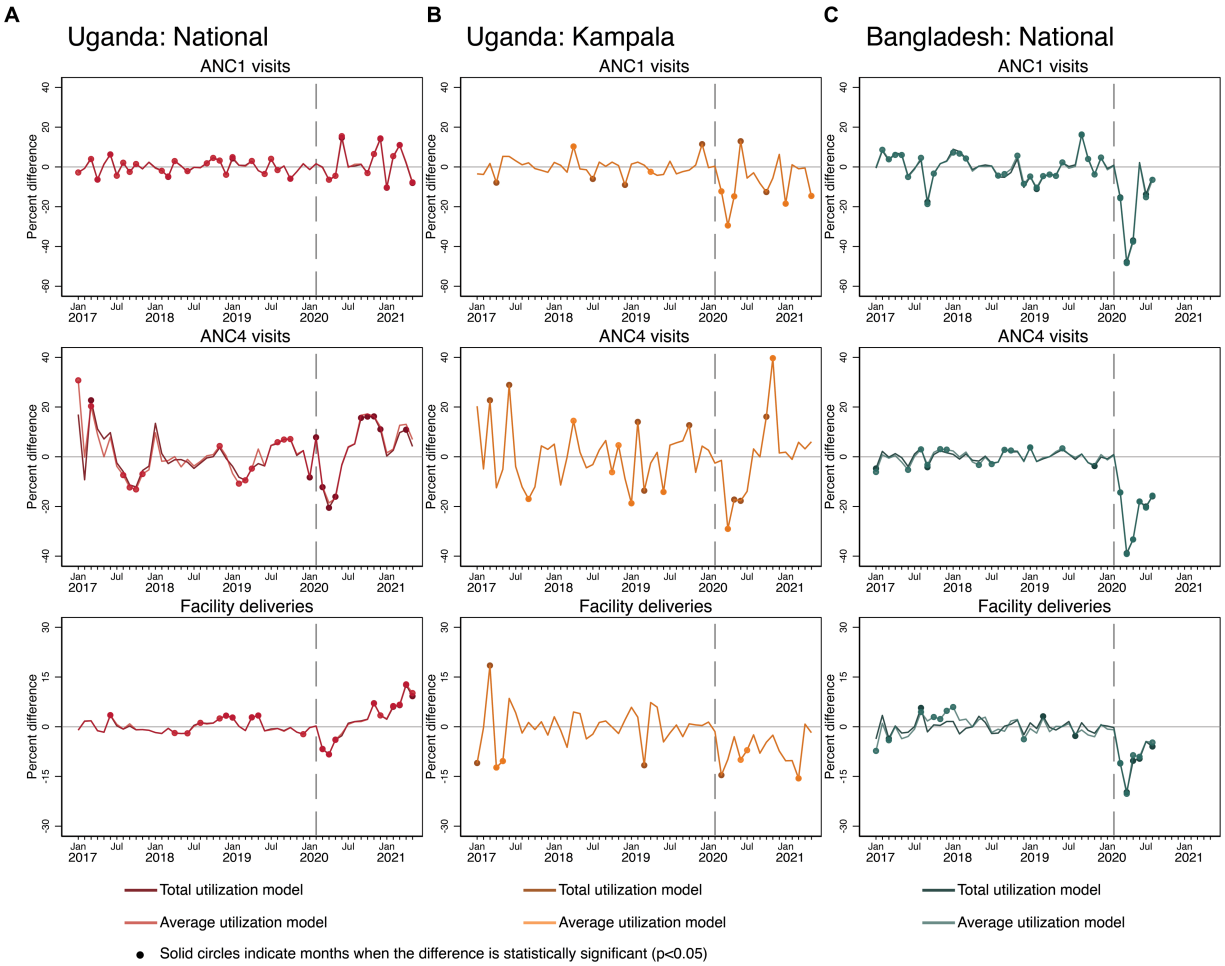
Estimated COVID-19 effects on maternal and newborn health services in Uganda and Bangladesh **(A)** Uganda: National, **(B)** Uganda: Kampala, and **(C)** Bangladesh: National.

The effects of COVID-19 on maternal and infant health services varied across countries (see Figure 4). In Uganda, the total and average ANC1 visits models show oscillating COVID-19 effects. Some months were significantly lower than predicted, and some months were significantly higher. This volatility in terms of maximum and minimum differences between observed and predicted values extends beyond the range of variability observed during the pre-COVID-19 months, suggesting that the instability is due to COVID-19. For ANC4, there are negative COVID-19 effects seen in the first 3 months of the pandemic in total and average visits, and it does appear that ANC4 visit levels recovered from June 2020 onward. Such patterns are even more pronounced when looking at the graphs for ANC specific to Kampala. For facility deliveries, at the national level, there are small initial, but significant negative COVID-19 effects in both models in March–May 2020, which then recovers and exceeds predicted levels by November 2020 onward, approximately 8–9 months after the pandemic began. Kampala shows a different pattern: there are negative COVID-19 effects in Kampala all throughout the COVID-19 months with just a small sign of recovery at the end of the time period.

In Bangladesh, there were severe negative COVID-19 effects for both total and average levels of ANC1 and ANC4 visits and facility deliveries. With the exception of 1 month of the first ANC visits, there was no consistent recovery in the levels of maternal and infant health services by August 2020 in Bangladesh.

### 3.4. Effects of COVID-19 on child health services

**Figure 5:**
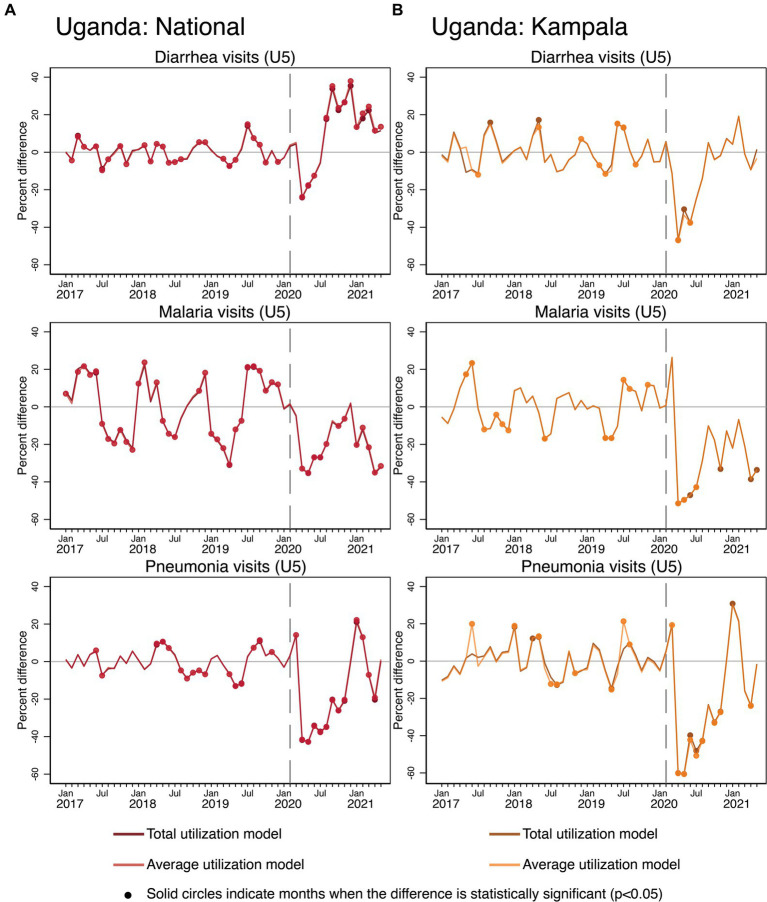
Estimated COVID-19 effects on the management of common childhood illnesses in Uganda **(A)** National, **(B)** Kampala.

There were consistent negative impacts of COVID-19 on illness-specific visits among children under five in Uganda only (see Figure 5). Equivalent data were not available in the MIS3 dataset for Bangladesh. Nationally, there were negative COVID-19 effects on diarrhea visits in April–June 2020, and higher-than-predicted levels from August 2020 onward for both average and total models. There were also strong negative effects on malaria and pneumonia-related visits among young children in Uganda in terms of the total and average models. However, for malaria, there is large and significant variability in the pre-COVID-19 period which indicates that the models did not replicate the observed service trajectories well. However, the entire curve of COVID-19 effects is in negative values after March 2020, except for 1 month, suggesting persistent COVID-19 effects. Levels of pneumonia-related visits in Uganda exceeded expected levels only in January and February 2021. In Kampala, there were also initial negative COVID-19 effects that were more severe compared to what occurred nationally for all three illnesses. Later patterns in the pandemic period in Kampala resembled national patterns except for diarrhea-related visits. In Kampala, diarrhea-related visits among young children met predicted values from July 2020 onward.

### 3.5. Effects of COVID-19 on childhood immunization

**Figure 6:**
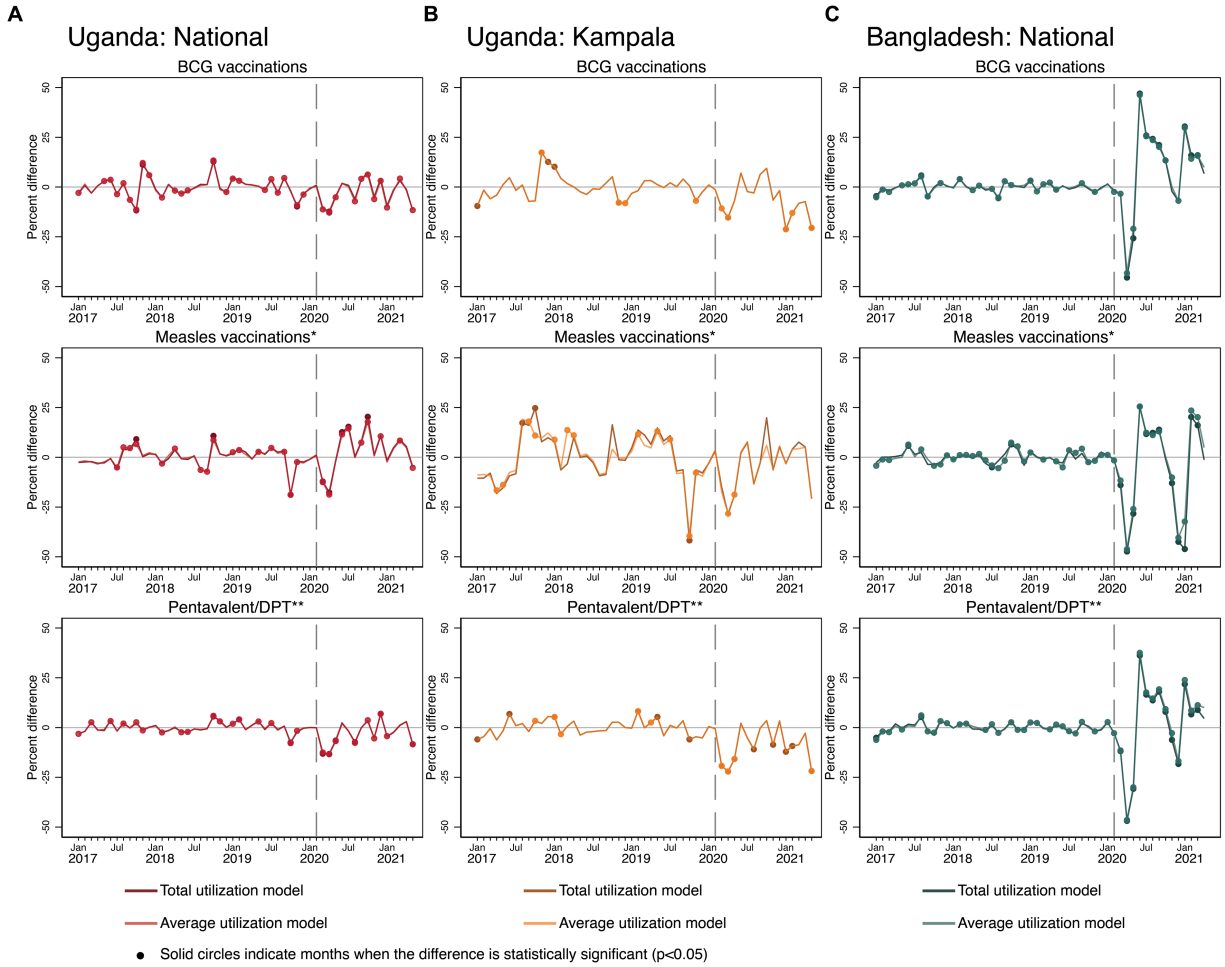
Estimated COVID-19 effects on childhood immunization in Uganda and Bangladesh **(A)** Uganda: National, **(B)** Uganda: Kampala, and **(C)** Bangladesh: National. Measles vaccinations in Uganda reflect any single dose administered, while in Bangladesh reflect the first of two doses of measles-rubella vaccinations. Pentavalent/DPT vaccinations in Bangladesh reflect first pentavalent doses. In Uganda, values reflect first DPT doses.

In terms of child vaccinations (see Figure 6), in Uganda, all three vaccines saw temporary negative COVID-19 effects at the beginning of the pandemic in March and April 2020. BCG and DPT vaccination levels showed some signs of recovery but alternated between higher and lower than expected levels throughout the entire rest of the time series. The measles vaccination pattern in Uganda had a similar shape in the pandemic period but alternated between achieving and exceeding expected levels from June 2020 onward. Recovery was achieved for all three vaccination types; however, it was only consistently sustained for measles vaccinations. In Kampala specifically, similar negative COVID-19 effects were detected compared with national patterns. However, for BCG and DPT vaccinations in Kampala, the graphs indicate significant negative and persistent COVID-19 effects toward the end of the time series.

In Bangladesh, pandemic period patterns were similar across vaccinations. There were large negative COVID-19 effects at the beginning of the pandemic, through May 2020. For all three vaccines, levels all recovered and exceeded predictions by June 2020. At the end of 2020, levels started to decline again, and were significantly lower than expected for all three vaccines by December 2020, but then quickly recovered by February 2021.

### 3.6. Effects of COVID-19 on family planning

**Figure 7:**
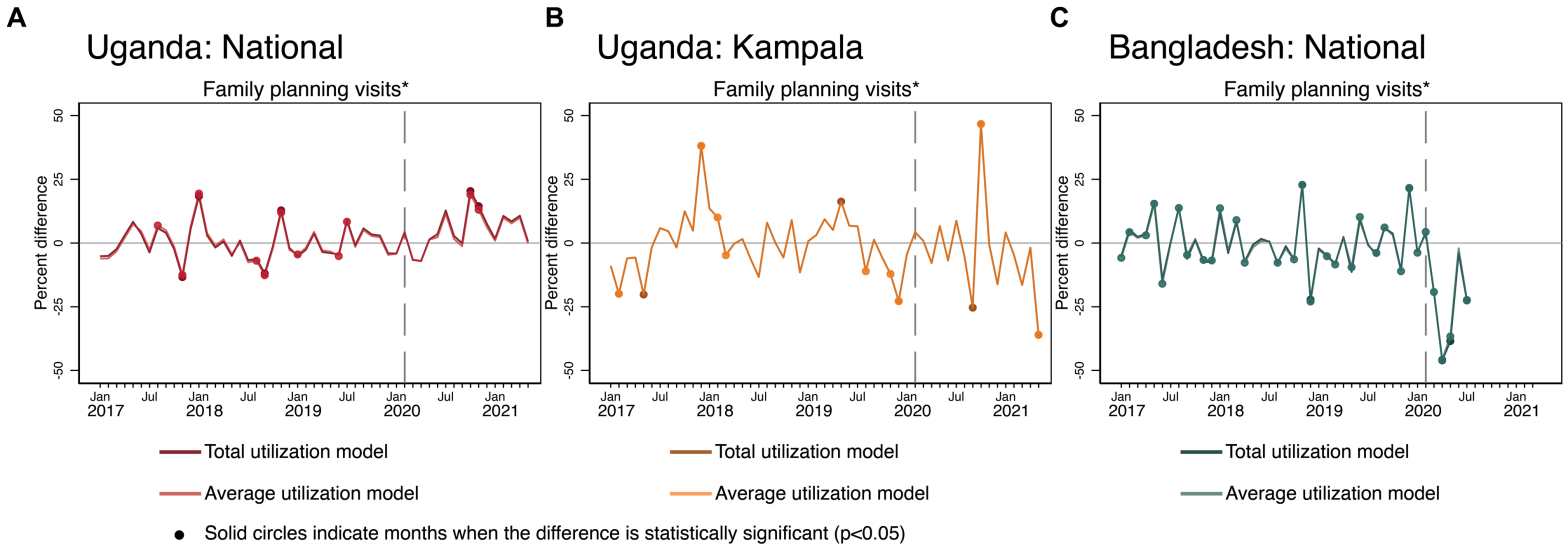
Estimated COVID-19 effects on FP in Uganda and Bangladesh **(A)** Uganda: National, **(B)** Uganda: Kampala, and **(C)** Bangladesh: National. FP visits in Uganda did not reflect all family planning methods but were calculated as the sum of visits for implants, oral contraceptives, condoms, and injectables.

Figure 7 shows the results on overall FP visits. There was a sharp negative COVID-19 effect in Bangladesh, while nationally in Uganda, there was no noticeable initial COVID-19 effect. In Kampala, there was more volatility during the pandemic period compared to the pre-COVID-19 period. Kampala experienced negative COVID-19 effects on FP services in September 2020, immediately followed by much higher than predicted levels in October.

In summary, COVID-19 had a statistically significant negative effect on the utilization of maternal health, FP, outpatient, and childhood vaccination services in Uganda and Bangladesh. The declines in service utilization levels were in the first few months of the COVID-19 pandemic, during the height of the lockdown period. By the end of the study periods (i.e., May 2021 for Uganda and August 2020 for Bangladesh’s MIS3 and April 2021 for Bangladesh’s EPI data), facility delivery and diarrhea treatment visits (for children aged 28 days to 5 years) recovered nationally in Uganda, but there was no consistent recovery for any of the MCH services in Kampala. For Bangladesh, vaccination services were the first to recover and exceeded pre-pandemic estimate levels by April 2021. Table 2 summarizes whether COVID-19 had an effect on utilization of MCH health services in the two countries.

**Table 2:** Summary of COVID-19 effects on health service utilization, Uganda and Bangladesh.

MCH services	Uganda (National)	Uganda (Kampala)	Bangladesh (National)
New outpatient visits (overall attendance)	Yes	Yes	Yes
1st antenatal visits	No	Yes	Yes
4th antenatal visits	Yes	Yes	Yes
Facility deliveries	Yes	Yes	Yes
Family planning visits*	No	No	Yes
Diarrhea treatment visits for children	Yes	Yes	--
Malaria treatment visits for children	Yes	Yes	--
Pneumonia treatment visits for children	Yes	Yes	--
BCG doses	No	Yes	Yes
Measles doses	Yes	Yes	Yes
1st DPT doses	Yes	Yes	Yes

BCG: Bacillus Calmette–Guérin vaccine; DPT: diptheria, pertussis, and tetanus toxoids vaccine. *Uganda family planning data is a composite of the most common methods that were consistent across time, which include male and female condoms, injectables, implants, and oral contraceptives. Other methods such as IUDs, emergency contraceptives, and natural methods were not included. **Bangladesh family planning data available ends in July 2020.

## 4. Discussion

In this analysis, we investigated two research questions on the effects of COVID-19 on health systems in Uganda and Bangladesh. We aimed to understand how COVID-19 affected reporting in routine information systems and how COVID-19 affected health service utilization.

For the first research question, we found that COVID-19 did not affect reporting for any services in Uganda and for the majority of health services in Bangladesh (see Figure 2). For immunization services in Bangladesh, we observed small, transitory negative effects on reporting for BCG and measles vaccines only (reporting of the pentavalent vaccine was unaffected). Other research has found negative effects of COVID-19 on facility-level reporting. Shapira and colleagues found drops in facility-level reporting for four of eight countries included in their analysis during COVID-19 (7). A recent study using data from the Democratic Republic of the Congo also found no COVID-19 effects on reporting for most of the basic services examined at the national level (29). In our study, we only used aggregate-level data (districts in Uganda and upazilas in Bangladesh). We acknowledge the possibility that COVID-19 effects could have occurred at the facility level but with the data we had available for our analysis, we were not able to detect that (i.e., even if a facility reported just one service provision, it would be recorded in the number of geographic units reporting service provision to RHIS). The transitory negative effects on reporting for immunization services in Bangladesh can possibly be due to disruptions in EPI outreach facilities, since the data from the EPI included monthly reporting from health facilities and EPI sites/satellite clinics in rural areas and NGOs in urban areas.

For the second research question, we found that health service utilization was affected by COVID-19 in both countries. For many of the services, the most severe impacts were experienced within the first 3 months of the pandemic, corresponding with the same time during which most governments implemented shutdown policies to prevent spread. Overall outpatient attendance as well as malaria and pneumonia visits among young children were the services most persistently negatively affected by COVID-19 nationally during the pandemic period and lacked stable recovery to expected levels. These results reflect existing studies that found negative impacts on overall utilization (4–8), as well as disease-specific service utilization (4, 6, 30). The pandemic has strained healthcare systems worldwide, redirecting resources and workforce toward COVID-19 response, resulting in reduced access to essential health services (11, 31). Fear of contracting the virus also has discouraged people from seeking healthcare services, leading to declines in facility-based care (31). In Nigeria, a study conducted in 2020 found that the pandemic led to a significant decline in the utilization of essential reproductive, maternal, and child health services due to area-wise lockdowns, unavailability of transports, and non-functioning health facilities (32). Also, in Pakistan and Iran, studies conducted in 2019–2021 reported that the pandemic resulted in a substantial decline in the utilization of essential health services resulting from lockdown measures and fear of exposing mothers and children to COVID-19 infection (14, 33, 34).

COVID-19 effects on maternal and infant health services were not consistent across countries. In Bangladesh, there were clear negative effects on ANC1, ANC4, and facility deliveries. There were some initial negative COVID-19 effects in Uganda, but after May 2020, there were multiple months exceeding levels predicted by our models. These inconsistent findings across countries have also been found in other studies. Research from Rwanda found significant decreases in ANC visits, facility deliveries, and live births nationally (18). A study from Ethiopia found month-to-month comparisons of ANC1 and ANC4 levels before and during COVID-19 to be similar (4). There are several possible explanations for the observed differences in the effect of COVID-19 on essential maternal health services utilization between Uganda and Bangladesh. First, the overall use of health facilities for delivery in Uganda is about 26 percentage points higher than that of Bangladesh (76.5% vs. 50.6% in 2016–2017), indicating that ANC and facility deliveries are still not perceived as essential in Bangladesh (35). Second, curfews and closure of public transport as a part of the national lockdown measures were enforced strictly in Uganda, unlike in Bangladesh, which affected a large proportion of patients relying on public transport to access healthcare facilities (36, 37).

During the COVID-19 period, supply-based services, which include family planning and vaccinations, were more volatile than what models had predicted. The pandemic’s impact on global healthcare systems and supply chain management has been profound—the outbreak of COVID-19 created one of the most vital supply chain disruptions of the past 50 years (38). A number of major healthcare facilities (e.g., distribution centers, factories, and warehouses) were situated in COVID-19 quarantine zones in China, Italy, India, the United States, Japan, Russia, and Australia (39), which resulted in a severe scarcity of critical medical supplies. There were initial negative COVID-19 effects for the first several months in Uganda and Bangladesh on immunization services, which is consistent with several other studies (7, 12, 18). Both countries either periodically achieved recovery or exceeded expected levels for multiple months in the remaining COVID-19 period, during months that had not been included in those other studies. A global modeling study also found that the second half of 2020 showed signs of recovery in childhood immunization in many countries, as global immunization rates began nearing expected estimates by December 2020 (40).

It is also important to note the drastic negative COVID-19 effects in Bangladesh on immunization services that occurred at the end of 2020. During this time, there was a health worker strike, particularly among the health workers whose responsibility it is to administer and implement national immunization programs. This drop in measles vaccinations is particularly prominent because this strike immediately preceded a national measles vaccination campaign (41). Due to a nationwide campaign, measles vaccinations that occurred in January 2021 were reported to a separate reporting system that was not integrated with the routine EPI information system.

When comparing national COVID-19 effect patterns in Uganda to those in Kampala the COVID-19 effects on Kampala were more severe. This pattern was consistent across almost every service investigated in this study. Relative to what occurred nationally, the increased severity in terms of COVID-19 effects presented as both larger gaps between observed and predicted values toward the beginning of the pandemic as well as delayed recovery later into the pandemic period in Kampala. This finding is not surprising as urban areas had COVID-19 outbreaks first and were often targeted for more stringent lockdown measures. Thus, the effects of the disruptions and lockdowns are likely to be more exacerbated in capital cities such as Kampala.

To address the adverse impacts of the COVID-19 pandemic on the use of essential health services, the governments of Uganda and Bangladesh adopted distinct strategies. In Uganda, several measures were initiated at the community, facility, district, and national levels to ensure uninterrupted health services during the pandemic (42). Beyond the rollout of a national strategy to maintain essential health services at the national level (43), these initiatives encompassed adaptations in community outreach services—such as training mothers to act as community liaisons encouraging others to utilize reproductive services and establishing a call center to aid in transporting clients to facilities amidst travel bans. These efforts aim to ensure that health services reach those beyond the facility settings. Health facilities have started using tents to mitigate overcrowding and innovatively repurposed their spaces to enable social distancing. There’s also been a shift in roles within health facilities, with nurses now monitoring women during labor and midwives dispensing medication, accommodating the increased patient load. Furthermore, district-level changes, like relocating health workers to the nearest facilities to their residences, granting vehicle permits for healthcare professionals during lockdowns, and deploying ambulances for expectant mothers requiring urgent care, have been put in place to tackle transportation issues (42). In Bangladesh, the government rolled out a national preparedness and response plan for COVID-19 and a national guideline for providing essential maternal, newborn, and child health services in the context of COVID-19 in March and May 2020, respectively. The Ministry of Health organized online training sessions with district-and facility-level health managers and face-to-face sessions with frontline healthcare workers providing MCH services to overcome the service provision bottlenecks and operational challenges during the pandemic (44). However, studies indicate that the government’s lockdown measures were not imposed effectively in rural areas and the movement restrictions gradually weaned off without proper guidelines or arrangements for scientific basis (44, 45). There was also a lack of coordination between various government entities in addressing emergency healthcare and on-the-ground crisis management, leading to severely compromising the availability of essential health services during the pandemic (37, 45).

This study has several strengths and further contributes to the expanding body of literature on the impact of COVID-19 on health service utilization from low and middle-income countries. The inclusion of data from two different countries allows for conclusions to be drawn across borders, while also illustrating the variety of ways COVID-19 could impact both reporting and service utilization. This research expands upon existing work that investigates similar questions. For almost all services in these countries, we predicted COVID-19 effects for at least 1 year after the start of the pandemic. Additionally, while many studies used interrupted time series approach using RHIS data to model changes in health service utilization due to disease outbreaks and government responses to those outbreaks, even before the onset of COVID-19 (7, 10, 17, 46, 47), we use rigorous, but simple modeling and prediction estimation strategies by adapting the interrupted time series approach that consider time trends, seasonal patterns, and unit-level fixed effects. Our models are easily replicable as they do not depend on information found outside the RHIS and can be implemented by widely available statistical software. Instead of very sophisticated, novel statistical modeling, we applied a general estimation strategy with the aim for wider use of such analytical approaches on routinely available data. We, however, found that the service delivery trajectories varied considerably by country and services, which requires analysts to be careful adapting the estimation model specification to the peculiarities of each specific service.

There are several limitations to our analysis pertaining to the models that we use to estimated COVID-19 effects. As time increases, the time trend estimated from pre-COVID-19 data becomes less relevant, and thus, the potential for error in the counterfactual prediction increases. Such inaccuracies could stem from not just coefficient inaccuracy, but also changes in model functional form. An analyst should be careful about the uncertainly of medium-and long-term predictions.

Bangladesh’s routine information system for maternal and child health services delivery expanded during the pre-COVID-19 period (in January 2019). Such changes were accounted for using the yearly control variables. In Uganda, the DHIS2 also changed in 2019, which had a new reporting mechanism and service definitions. However, we found consistency in the time series across years. Finally, we want to mention that adjusting for COVID-19 effects on reporting in total models influenced the conclusions drawn from this analysis. Had we not considered the potential impact of COVID-19 on reporting for EPI services in Bangladesh, we may have misestimated COVID-19 effects for some services.

## 5. Conclusion

Our analysis indicates the COVID-19 pandemic affected the health service utilization for essential health services in Uganda and Bangladesh. Which services were affected, the magnitude of these effects, and the recovery patterns varied across and within countries. Overall attendance, and visits among children under 5 for malaria and pneumonia were most persistently affected during the first year of the pandemic. Services in Bangladesh were universally and severely affected by COVID-19, with additional severe impacts later in the year on vaccination services due to health workforce strikes. Delayed negative impacts and persistent instability were also seen in Uganda.

We used an adaptation of a well-validated approach to model changes in health service utilization due to disease outbreaks such as COVID-19 pandemic, and our approach is feasible in terms of data requirement and availability of statistical software. The analytical approach used in this paper could be very useful as a form of surveillance for health services disruptions from any cause (such as strikes or disasters/floods or outbreaks) leading to rapid responses from health service managers and policymakers. Government agencies may consider including the type of analysis presented in this paper as a routine practice for assessing their provision of essential health services.

## Data availability statement

The datasets presented in this study can be found in online repositories. Routine health information data used for this analysis are open for public access using the data portals of the respective government agencies. For Bangladesh, the data is available from the Directorate General of Family Planning’s Management information system’s website (https://dgfpmis.org/). For Uganda, data are available from the Ministry of Health’s DHIS2 system (http://hmis2.health.go.ug/). The datasets developed and analyzed during the current study is available in the USAID repository, Dataverse, at the following link: https://dataverse.unc.edu/dataset.xhtml?persistentId=doi:10.15139/S3/30EWWK.

## Ethics statement

The studies involving humans were conducted according to the guidelines of the Declaration of Helsinki and approved by the Office of Human Research Ethics (OHRE) of the University of North Carolina at Chapel Hill. The studies were conducted in accordance with the local legislation and institutional requirements. Written informed consent for participation was not required from the participants or the participants’ legal guardians/next of kin in accordance with the national legislation and institutional requirements.

## Author contributions

GA and WW conceptualized the analyses, which were done by them and HS. MK and NR prepared the Bangladesh data and analysis of contextual factors with assistance from KA. KS, JM, and ES prepared the data from Uganda. HS, GA, and KA produced the first draft of the manuscript, which was reviewed by GE, MK, NR, KS, JM, ES, and WW. KA had final responsibility for the decision to submit for publication. All authors contributed to subsequent drafts and approved the final version for publication.
