# Supplementary material for: Estimating the effects of COVID-19 on essential health services utilization in Uganda and Bangladesh using data from routine health information systems
Source: Front Public Health. 2023 Sep 27;11:1129581. doi: 10.3389/fpubh.2023.1129581 (PMC10564984; doi:10.3389/fpubh.2023.1129581)
Supplement: Supplementary file 1 [file Table_1.DOCX]

Supplementary Material

Estimating the effects of COVID-19 on essential health services utilization in Uganda and Bangladesh

Gustavo Angeles, Hannah Silverstein, Karar Zunaid Ahsan*, Mohammad Golam Kibria, Nibras Ar Rakib, Gabriela Escudero, Kavita Singh, Jamiru Mpiima, Elizabeth Simmons, William Weiss

*** Correspondence:** Corresponding Author: zunaid@email.unc.edu

# Determining the functional form of time in the estimation model

We began by graphing the time series of each service to get a sense of the overall patterns of service utilization over time. We then examined changes between adjacent time points, which we called “first differences,” and the changes in those first differences between adjacent time points, which we called “second differences.” We graphed the first and second differences and examined their averages and standard deviations.

Examining first and second differences is a way to analyze the first and second derivatives of a function. This is very informative for determining the functional form of the relationship between use of services and time: If the actual functional form is quadratic on time, then it would have the following expression:

$Y_{t}=\alpha_{0}+\alpha_{1}{time}_{t}+\alpha_{2}{time}_{t}^{2}$ (1)

where $Y_{t}$ is the service outcome of interest and ${time}_{t}$ is the continuous count of time up to *t*. The first derivative of equation (1) would be $\frac{dY}{dtime}=Y^{'}=\alpha_{1}+2\alpha_{2}time$. The second derivative would be $\frac{d^{2}Y}{d{time}^{2}}=Y^{''}=2\alpha_{2}$, with $\alpha_{2}\neq0$.

Instead, if the actual functional form is linear on time, then it would have the following expression:

$Y_{t}=\alpha_{0}+\alpha_{1}{time}_{t}$ (2)

which has as first derivative: $Y^{'}=\alpha_{1}$, a constant term, and as second derivative: $Y^{''}=0$.

Therefore, to determine which functional form to use, quadratic or linear, we can just examine the first and second derivatives according to these rules:

- If the second derivative is zero, and the first derivative is a non-null constant, then the functional form to use is linear on time.
- If the second derivative is a non-null constant, and the first derivative changes linearly over time, then the functional form is quadratic on time.

We examined the empirical first and second differences of the time series for each service and country and concluded that for all services the linear functional form was linear, not quadratic.

# Assessing model fit

To assess model fit we considered the models’ $R^{2}$, adjusted-$R^{2}$, and root-mean-square error (RMSE) for the reporting and total service utilization models. We also generated predicted values for each pre-COVID-19 month and examined how well the predicted trajectory replicated the observed trajectory of services. We graph the predicted and observed time series and inspected how well the predicted series tracked the level, trend, seasonality and volatility of the series over time. In addition, we examined the average difference between $\hat{y_{i}}$ predicted and $y_{t}$ observed average values in the numerator, using the following measure-of-fit:

*adapted RMSE* $=\sqrt{\frac{\sum_{t=1}^{38} {(\hat{y_{t}}-y_{t})}^{2}}{38}}$.
